# Symptom Characterization and Outcomes of Sailors in Isolation After a COVID-19 Outbreak on a US Aircraft Carrier

**DOI:** 10.1001/jamanetworkopen.2020.20981

**Published:** 2020-10-01

**Authors:** Gadiel R. Alvarado, Benjamin C. Pierson, Eric S. Teemer, Hector J. Gama, Ronald D. Cole, Samuel S. Jang

**Affiliations:** 1 Infectious Disease Department, Brooke Army Medical Center, Fort Sam Houston, Texas; 2 US Army Medical Research Institute of Infectious Diseases, Frederick, Maryland; 3 Public Health Department, D. D. Eisenhower Army Medical Center, Fort Gordon, Georgia; 4 Army Public Health Center, Aberdeen Proving Ground, Maryland

## Abstract

This case series describes the symptoms and outcomes of sailors during and after an outbreak of coronavirus disease 2019 (COVID-19) on the USS Theodore Roosevelt.

## Introduction

Reports of severe acute respiratory syndrome coronavirus 2 (SARS-CoV-2) outbreaks affecting nursing homes, homeless shelters, and cruise ships describe both asymptomatic and symptomatic cases among patients whose primary risk factor for acquisition was residence in a confined congregate environment.^1,2,3^ However, the age distribution of patients with coronavirus disease 2019 (COVID-19) described is weighted heavily toward elderly individuals and those with preexisting conditions. The USS Theodore Roosevelt (TR) outbreak investigation by the US Navy and Centers for Disease Control and Prevention illuminated how the virus affects a young military population.^4^ In this study, the US Army Public Health COVID-19 Task Force describes the results of an independent investigation of the shore-based USS TR outbreak response and 736 USS TR sailors in isolation status.

## Methods

This study was approved and granted a waiver of consent by the US Navy Bureau of Medicine and Surgery; consent was waived because the study was deemed to post minimal risk to the participants. This study follows the reporting guideline for case series.

 This case series included all USS TR sailors with a diagnosis of SARS-CoV-2 infection and placed in isolation at Naval Base Guam between March 31 and April 15, 2020. Polymerase chain reaction tests for COVID-19 (BioFire Respiratory Panel With SARS-CoV-2, bioMérieux) were performed through nasal swabs for all sailors. Sailors with a diagnosis of COVID-19 were isolated. Those who tested negative for SARS-CoV-2 and those who were asymptomatic were quarantined in single-room hotel accommodations.

Daily monitoring was performed by clinical staff through face-to-face evaluations using standardized questionnaires. Sailors who developed symptoms during quarantine were retested and moved to isolation. A polymerase chain reaction test–based deisolation strategy was used for all sailors. Demographic and epidemiological characteristics and clinical and laboratory data were reviewed and analyzed.

Sailors who reported any symptom throughout the study period were characterized as symptomatic, and each symptom category was described. An epidemiological curve was created using data from 218 sailors for whom symptom onset date was documented. Data analysis was performed using R statistical software version 3.6.3 (R Project for Statistical Computing) from March to April 2020.

## Results

Of 4085 USS TR sailors who disembarked, 736 had a diagnosis of SARS-CoV-2 (median age, 25 years; interquartile range, 22-31 years; 572 men [77.7%]). Five hundred ninety sailors (80.2%) were characterized as symptomatic, with a median symptom duration of 7 days (interquartile range, 5-11 days). One hundred forty-six sailors (19.8%) remained asymptomatic for the duration of the study period. Cough was observed for 677 person-days (13.6%), coldlike symptoms for 483 person-days (9.7%), anosmia for 463 person-days (9.3%), headache for 438 person-days (8.8%), ageusia for 393 person-days (7.9%), and fever for 65 person-days (1.3%). With regard to clinical outcomes, 729 sailors remained in outpatient isolation, 6 were hospitalized, and 1 died during the study period (Table). An epidemiological curve is shown in the Figure. As shown in the Figure, the peak of the outbreak occurred on March 30, with 30 new cases.

**Table.** Characteristics of USS Theodore Roosevelt Sailors With Severe Acute Respiratory Syndrome Coronavirus 2 Infection

Characteristic	Sailors, No. (%) (N = 736)	Person-days, No. (%) (N = 4976)
Age, median (interquartile range), y	25 (22.0-31.0)	
Sex		
Male	572 (77.7)	
Female	164 (22.3)	
Symptoms^a^		
Symptomatic	590 (80.2)	
Asymptomatic	146 (19.8)	
Duration, median (interquartile range), d	7 (5-11)	
Cough	332 (45.1)	677 (13.6)
Coldlike symptoms^b^	386 (52.5)	483 (9.7)
Anosmia	275 (37.4)	463 (9.3)
Headache	252 (34.2)	438 (8.8)
Ageusia	230 (31.3)	393 (7.9)
Sore throat	195 (26.5)	338 (6.8)
Body aches	146 (19.8)	244 (4.9)
Dyspnea	23 (3.1)	104 (2.1)
Chest pain	87 (11.8)	104 (2.1)
Chills	52 (7.1)	90 (1.8)
Gastrointestinal symptoms^c^	65 (8.8)	70 (1.4)
Fever^d^	55 (7.5)	65 (1.3)
Fatigue	40 (5.4)	45 (0.9)
Clinical outcomes		
Outpatient isolation	729 (99.0)	
Hospitalized^e^	6 (0.9)	
Deaths	1 (0.1)	

^a^ Daily face-to-face screening and evaluation occurred March 31 through April 15, 2020.

^b^ Incudes nasal congestion, rhinorrhea, and/or sneezing.

^c^ Includes nausea, vomiting, diarrhea, and/or abdominal pain.

^d^ Defined as oral temperature greater than 100 °F.

^e^ Admission diagnoses included coronavirus disease 2019 pneumonia for 5 sailors and chest pain evaluation for 1 sailor.

**Figure.**
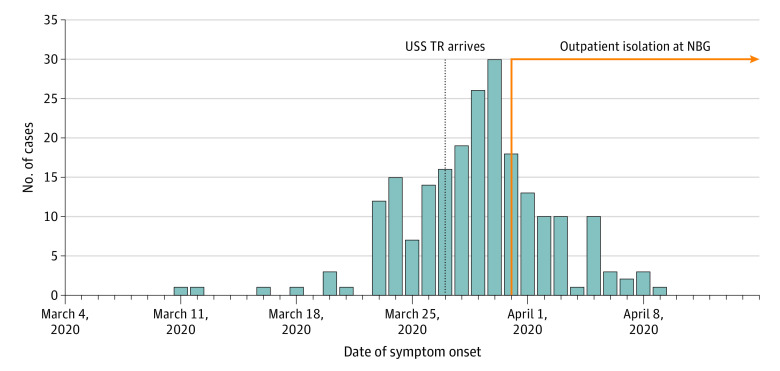
USS Theodore Roosevelt (TR) Coronavirus Disease 2019 Cases With Recorded Date of Symptom Onset NBG indicates Naval Base Guam.

## Discussion

The frequency of symptoms observed in this study is consistent with that in previous reports^5^ of COVID-19 among nonhospitalized patients. The person-days proportion analysis confirms that olfactory and gustatory symptoms are commonly seen in minimally symptomatic COVID-19.^6^ One hundred forty-six sailors (19.8%) remained asymptomatic for the duration of the study period, and this is consistent with the symptom survey results from the US Navy and Centers for Disease Control and Prevention report (18.5%),^4^ which also highlights the concern for enhanced asymptomatic SARS-CoV-2 transmission. Although this study is limited to the isolation group sailors during a specific period, many of the conclusions should be generalizable to the overall shipboard population.

In a confined space congregate setting with young essential workers, COVID-19 is unlikely to be clinically distinguishable from other acute respiratory illness without specific laboratory testing. Asymptomatic (and presymptomatic) spread will limit the effectiveness of symptomatic screening in the absence of other nonpharmaceutical interventions, such as testing, masking, and, as feasible, social distancing. Finally, the rapid increase in case number as incubating cases disembarked, followed by the precipitous decrease in cases, suggests that the shore-based nonpharmaceutical interventions interrupted a probable acceleration in case incidence that would have likely resulted in a substantial disease burden. Lessons learned from the USS TR COVID-19 outbreak may have applicability in other congregate settings staffed by essential workers and in understanding clinical features of the illness in younger adult populations.
