# Error in Methods

**DOI:** 10.1001/jamanetworkopen.2020.28432

**Published:** 2020-12-16

In the Research Letter titled “Symptom Characterization and Outcomes of Sailors in Isolation After a COVID-19 Outbreak on a US Aircraft Carrier,”^1^ published October 1, 2020, the authors have requested a correction to the approval statement in the first sentence of the Methods section. The corrected statement is, “The Navy Bureau of Medicine and Surgery cleared this document for publication for release under Authored Works guidelines, which included a waiver of institutional review board approval for this secondary analysis and indication that this report met requirements of the Health Insurance Portability and Accountability Act privacy rules.” This article has been corrected.^1^
